# Osmotic Adjustment in Wheat (*Triticum aestivum* L.) During Pre- and Post-anthesis Drought

**DOI:** 10.3389/fpls.2022.775652

**Published:** 2022-01-31

**Authors:** Sarah Verbeke, Carmen María Padilla-Díaz, Geert Haesaert, Kathy Steppe

**Affiliations:** ^1^Laboratory of Plant Ecology, Department of Plants and Crops, Faculty of Bioscience Engineering, Ghent University, Ghent, Belgium; ^2^Department of Plant Envirogenetics, Faculty of Science and Engineering, Maastricht University, Maastricht, Netherlands; ^3^Department of Plants and Crops, Faculty of Bioscience Engineering, Ghent University, Ghent, Belgium

**Keywords:** plant stress, leaf water potential, source-sink, turgor, *Van't Hoff* equation, osmotic adjustment, osmotic potential, carbohydrate mobilization

## Abstract

Pre-anthesis drought is expected to greatly increase yield losses in wheat (*Triticum aestivum* L.), one of the most important crops worldwide. Most studies investigate the effects of pre-anthesis drought only at maturity. The physiology of the plant before anthesis and how it is affected during drought is less studied. Our study focused on physiological patterns in wheat plants during pre- and post-anthesis drought. To this end, we measured leaf xylem water potential, osmotic potential and water content in different plant parts at a high temporal frequency: every 3 days, three times a day. The experiment started just before booting until 2 weeks after flowering. Drought stress was induced by withholding irrigation with rewatering upon turgor loss, which occurred once before and once after anthesis. The goal was to investigate the patterns of osmotic adjustment, when it is used for protection against drought, and if the strategy changes during the phenological development of the plant. Our data gave no indication of daily osmotic adjustment, but did show a delicate control of the osmotic potential during drought in both leaves and stem. Under high drought stress, osmotic potential decreased to avoid further water loss. Before anthesis, rewatering restored leaf water potential and osmotic potential quickly. After anthesis, rewatering restored water potential in the flag leaves, but the osmotic potential in the stem and flag leaf remained low longer. Osmotic adjustment was thus maintained longer after anthesis, showing that the plants invest more energy in the osmotic adjustment after anthesis than before anthesis. We hypothesize that this is because the plants consider the developing ear after anthesis a more important carbohydrate sink than the stem, which is a carbohydrate sink before anthesis, to be used later as a reserve. Low osmotic potential in the stem allowed turgor maintenance, while the low osmotic potential in the flag leaf led to an increase in leaf turgor beyond the level of the control plants. This allowed leaf functioning under drought and assured that water was redirected to the flag leaf and not used to refill the stem storage.

## 1. Introduction

Globally, wheat is contributing for 20% of the caloric and protein intake of the human population (Lobell and Gourdji, 2012; Shiferaw et al., 2013; Cosgrove, 2021). Food demand is expected to double by 2050 (Tilman et al., 2011). Meanwhile, climate change is not only endangering the global food productivity, but also local food security (Lobell and Gourdji, 2012). Drought affects 60% of the wheat production in high-income countries and 30% in least developed countries (Chen et al., 2012; Ahmad et al., 2018). Up to 70% of yield losses can occur due to drought (Nouri-Ganbalani et al., 2009). To keep up with the demand, strategies need to be developed to increase wheat yield under this changing environment (Ray et al., 2013; Hunter et al., 2017). This will require the collaboration of plant physiologists, plant breeders, geneticists, agronomists, computer scientists and more. There is a necessity to understand the physiology more in depth and how this physiology is impacted by drought stress. With a better understanding, crop management decisions can be improved or new targets for breeding can be discovered.

Drought affects wheat differently depending on the growth stage (Fischer, 1973; Villegas et al., 2001; Del Pozo et al., 2007; Tatar et al., 2016). Most of the research on drought is done at the grain filling stage, also named terminal drought or post-anthesis drought (e.g. Nicolas et al., 1985; Shah and Paulsen, 2003; Pradhan et al., 2012), since this stage is very drought sensitive. However, in Mediterranean areas, where a lot of the wheat cultivation is rain-fed (El Hafid et al., 1998; Tatar et al., 2016), dry periods are expected to increase (IPCC, 2018). So not only terminal drought will become a major yield limiting factor, but also drought during the elongation phase (or pre-anthesis drought). A considerable smaller amount of research is performed on pre-anthesis drought, even though it is an important stage to study drought stress. Since flower initiation takes place in this stage, some sources say it is more sensitive than the grain filling stage (Fischer, 1973; Tatar et al., 2016; Ahmad et al., 2018; Dietz et al., 2021). The known effects of pre-anthesis drought are a reduced height, tillering, leaf area, biomass and yield (e.g. Kadam et al., 2014; Mwadzingeni et al., 2016; Qaseem et al., 2019) and early flowering (Foulkes et al., 2007). Most sources report these post-anthesis effects of pre-anthesis drought, such as yield and water-use efficiency (e.g. Inoue et al., 2004; Mwadzingeni et al., 2016; Hlavacova et al., 2018; Qaseem et al., 2019; Lou et al., 2021). Yet the exact physiology of the plant during this stage is not as well characterized.

A few sources have measured some physiological parameters during the elongation phase. Some studies report on a reduced water content and water potential in this phase due to drought (Karamanos et al., 1983; Siddique et al., 2000; Inoue et al., 2004; Qaseem et al., 2019). Stomatal closure has also been observed and will then prevent the water potential to decline too quickly (Liang et al., 2002; Inoue et al., 2004; Monneveux et al., 2006; Subrahmanyam et al., 2006) and protect the plant against severe dehydration. Plants also have other avoidance and tolerance mechanisms to cope with drought stress (Blum, 1989), but these have rarely been measured in wheat during the elongation phase. Osmotic adjustment is such other protective mechanism. As the water content in the plant declines, compatible solutes such as proline (Karamanos et al., 1983; Nazarli and Faraji, 2011; Mwadzingeni et al., 2016; Qaseem et al., 2019), non-structural sugars (Ashraf and Foolad, 2005; Nazarli and Faraji, 2011), and other organic and non-organic solutes (Ahmad et al., 2018) are being imported into the cells to decrease the osmotic potential. This avoids more water loss. Turgor is thus maintained within the cells as long as possible, avoiding wilting and enabling growth (Ashraf and Foolad, 2005; Ahmad et al., 2018). Restoration of turgor allows the stomata to re-open partially under mild drought stress (Ahmad et al., 2018). Not much research was found on osmotic adjustment specifically in the elongation phase, but Karamanos et al. (1983) reported a differential increase in proline content due to drought stress when comparing before and after anthesis. This suggests a differential strategy of osmotic adjustment in the presence of the ear as a new sink.

The present study focused on drought-induced patterns of several physiological parameters in a phenological context. A drought sensitive wheat cultivar (*Triticum aestivum* L. cultivar Viking) was subjected to severe drought stress that started before booting and lasted 2 weeks after flowering. The plants were rewatered when they started visually wilting to maintain growth: once before and once after anthesis. Water content, leaf xylem water potential and osmotic potential were measured three times a day, every 3 days. By comparing the osmotic to the xylem water potential and water content, we could differentiate between a changing water content and a changing osmolyte content. By measuring the osmotic potential, instead of sugar or proline content alone, all osmolytes were taken into account and therefore represent the osmotic adjustment best. We wanted to assess when osmotic adjustment is occurring, specifically if there were diurnal patterns or phenological influences, and how it is affected by drought stress. Because the two rewatering events took place before and after flowering, we could examine if there was a difference in strategy when the developing ear was present as a new carbohydrate sink.

## 2. Materials and Methods

### 2.1. Plant Material and Environment

A drought sensitive spring wheat cultivar (*Triticum aestivum* L. cultivar Viking) was selected from a screening experiment (data not shown). The seeds were sown on June 3, 2020 (Days After Sowing, DAS 0) in 4 L pots filled with equal amounts of commercial potting mix (Structural nr1, Schebbout M., Kaprijke, Belgium: DM 30%, pH 5-6.5, EC 350 μS.cm^−1^ with NPK fertilizer 14-16-18 1.25 kg.m^−3^). In 56 pots, ten seeds per pot (*n* = 560) were sown at a depth of 3 cm and irrigated immediately with 100 mL water. Seed germination was over 90%. The pots were placed on two adjacent table beds in a growth chamber (WEKK 10.40.8L SN 40816000381001, Weiss Technik, Reiskirchen-Lindenstruth, Germany), where the environment was controlled. During the first 4 weeks of plant development (DAS 0-33), the temperature was set at 21/ 16 °C day/ night cycle (8 a.m. to 8 p.m.). The day was simulated by allowing the environment to linearly come to a temperature of 21 °C at 12 p.m. The temperature started declining at a constant rate again from 2 p.m. to reach a temperature of 16 °C at 8 p.m. After 4 weeks of growth (DAS 33; trefoil stage), the temperature was increased to a 23/ 17 °C day/ night cycle with a similar daytime increase. The plants were illuminated with artificial light (T5 Reflex Cool White, Philips, Eindhoven, Netherlands). The photoperiod was set at 12 h/ 12 h, matching the temperature. In the first 4 weeks the lamps were set at 200 μmol.m^−2^.s^−1^ during the day, with a temporary increase to 300 μmol.m^−2^.s^−1^ between 12 and 2 p.m. After 4 weeks this was increased to 300 and 400 μmol.m^−2^.s^−1^ respectively. The relative humidity (RH) was controlled between 40 and 60%, but data showed that RH reached 75% during the days and 90% during the nights.

Temperature and relative humidity were measured with a RH/T sensor (type EE08, E+E Elektronik, Engerwitzdorf, Austria) in the middle of each table bed. Photosynthetic Active Radiation (PAR) was measured with a quantum sensor (SQ-110-SS, Apogee Instruments, Logan, UT-US) also in the middle of each table bed, and atmospheric CO_2_ concentration was monitored with a carbon dioxide probe (CARBOCAP GMP343, Vaisala, Vanha Nurmijärventie, Finland). The sensors were placed at the height of the canopy. The data was logged every 2 min with a data logger (CR1000 and AM16/32 Multiplexer, Campbell Scientific, Logan, UT-US) and collected in the PhytoSense software (Phyto-IT, Ghent, Belgium).

### 2.2. Watering and Drought Treatment

The pots were randomly reorganized in the growth chamber every 2 weeks (on DAS 12, 26, 42 and 54) until the start of the drought treatment (on DAS 58) to avoid positional artifacts. To minimize inter-pot variability, the plants did not receive any fertilizer, nor were they treated with pesticides. No pests were detected during the experiment. Every 2–3 days the plants were irrigated manually at field capacity, which corresponded to a Volumetric Water Content (VWC) of 35%. This was confirmed by regular measurements with a soil moisture sensor (SM 300 Moisture Sensor and HH2 Moisture Meter, Delta-T Devices, Cambridge, UK). On DAS 54 (at flag leaf appearance), one bed, containing half of the pots (*n* = 280) was assigned to the control treatment and received irrigation to field capacity as described before. The other bed (*n* = 280) was assigned to the drought treatment and received irrigation at 50% field capacity on DAS 54 and 56 to initiate the drying process. By DAS 58, the drought treatment was started and irrigation was withheld. The drought stressed plants were irrigated to field capacity again on DAS 66 and 75, when the plants started wilting and turgor was lost, after which the drought treatment continued.

### 2.3. Measurements

Measurements were performed from the start of the drought treatment (DAS 58, 3 days after flag leaf appearance) until the end of the experiment (DAS 84, 10–12 days after flowering) every 3 days. Each measurement day constituted three measurement periods: predawn (6–7:30 a.m.), midday (12–1:30 p.m.) and afternoon (5–6:30 p.m.). The measurements were performed in five repetitions for each treatment and included: stem fresh and dry weight, xylem water potential of the bottom leaf and flag leaf and osmotic potential of the stem base and flag leaf. Plant material was weighed within 2 h after excision with a precision balance (ML104T/00, Mettler Toledo, Columbus, VS) and then dried at 60 °C for at least 7 days after which the dry weight was determined. For the water potential measurements, the leaves were excised and stored for a few minutes in an air tight doubled plastic bag with elevated humidity by enclosing a moist paper towel between the outer and inner bag and by breathing in the bag a few seconds before introducing plant material in the inner bag. This ensured equilibrium of any water potential gradient within the leaf (Trueba et al., 2019) and prevented dehydration (Corso et al., 2020). The leaf water potential was then measured with a Scholander pressure chamber (model 600, PMS Instrument Company, Albany, OR-USA). The osmotic potential was measured by cutting a small piece of the base of the stem, or the base of the flag leaf. These samples were flash frozen in liquid nitrogen and stored at –80° C until they were analyzed with a thermocouple psychrometer (HR 33T, Wescor, Logan, UT-US) according to the manufacturer's instructions. Leaf and stem samples were taken from the main shoots of the plants that were furthest in their development (while still omitting outliers) to limit the biological variation in phenology at each sampling.

### 2.4. Data Analysis

The data was processed and visualized in R. Relative water content (*RWC*, -) was calculated as


(1) $$\begin{array}{r} RWC=\frac{FW-DW}{FW} \end{array}$$

with *FW* the fresh weight in g and *DW* the dry weight in g. Since both the total (xylem) water potential and osmotic potential were measured in the flag leaf, the turgor could be calculated as the difference between these two variables. To visualize the osmotic adjustment, the osmolyte content was calculated according to the *Boyle - Van 't Hoff* equation (Nobel, 1969):


(2) $$\begin{array}{r} ROC=\frac{\psi_{\pi }RWC}{R_{g}T} \end{array}$$

with ψ_π_ (MPa) the osmotic potential, *R*_*g*_ the ideal gas constant (8.314 J.K^−1^.mol^−1^), *T* (K) the temperature in the psychrometer chamber, and *ROC* (mol.g^−1^) the relative osmolyte content, since the water volume is expressed relatively to the fresh weight. *ROC* is an approximation of the osmolyte content, since there is usually a need for a correction factor as the cellular content is not an ideal solution (Yokozeki, 2006).

After checking normality with the Shapiro-Wilk's test, Student *t*-tests were performed at each time point to detect significant differences between the control and drought treatment of the different variables. To detect significant trends over time, multiple linear regression analyses were performed for the xylem water potential (ψ_*x*_, MPa), osmotic potential (ψ_π_, MPa), relative water content in the stem (*RWC*, -) and turgor in the flag leaf (ψ_*p*_, MPa):


(3) $$\begin{array}{r} \psi_{x}=\psi_{soil}+DAS*Leaf+Time*Leaf \end{array}$$

(4) $$\begin{array}{r} \psi_{\pi }=\psi_{soil}+DAS*Organ+Time*Organ \end{array}$$

(5) $$\begin{array}{r} RWC=\psi_{soil}+DAS+Time \end{array}$$

(6) $$\begin{array}{r} \psi_{p}=\psi_{soil}+DAS+Time \end{array}$$

with ψ_*soil*_ the soil water potential in kPa, *DAS* the day after sowing, *Time* a dummy variable indicating the time of day when the measurements were taken (predawn, midday or afternoon) with midday as the base (to be able to easily compare the changes predawn-midday and midday-afternoon), *Leaf* a dummy variable indicating the bottom or flag leaf with the flag leaf as the base and *Organ* a dummy variable indicating the flag leaf or stem with the stem as the base. To detect daily osmotic adjustment, another regression analysis was performed linking the osmotic potential to the *RWC*:


(7) $$\begin{array}{r} \psi_{\pi }=RWC+Treatment*Time \end{array}$$

with *Treatment* a dummy variable indicating the control or drought treatment with the drought treatment as the base.

## 3. Results

**Figure 1:**
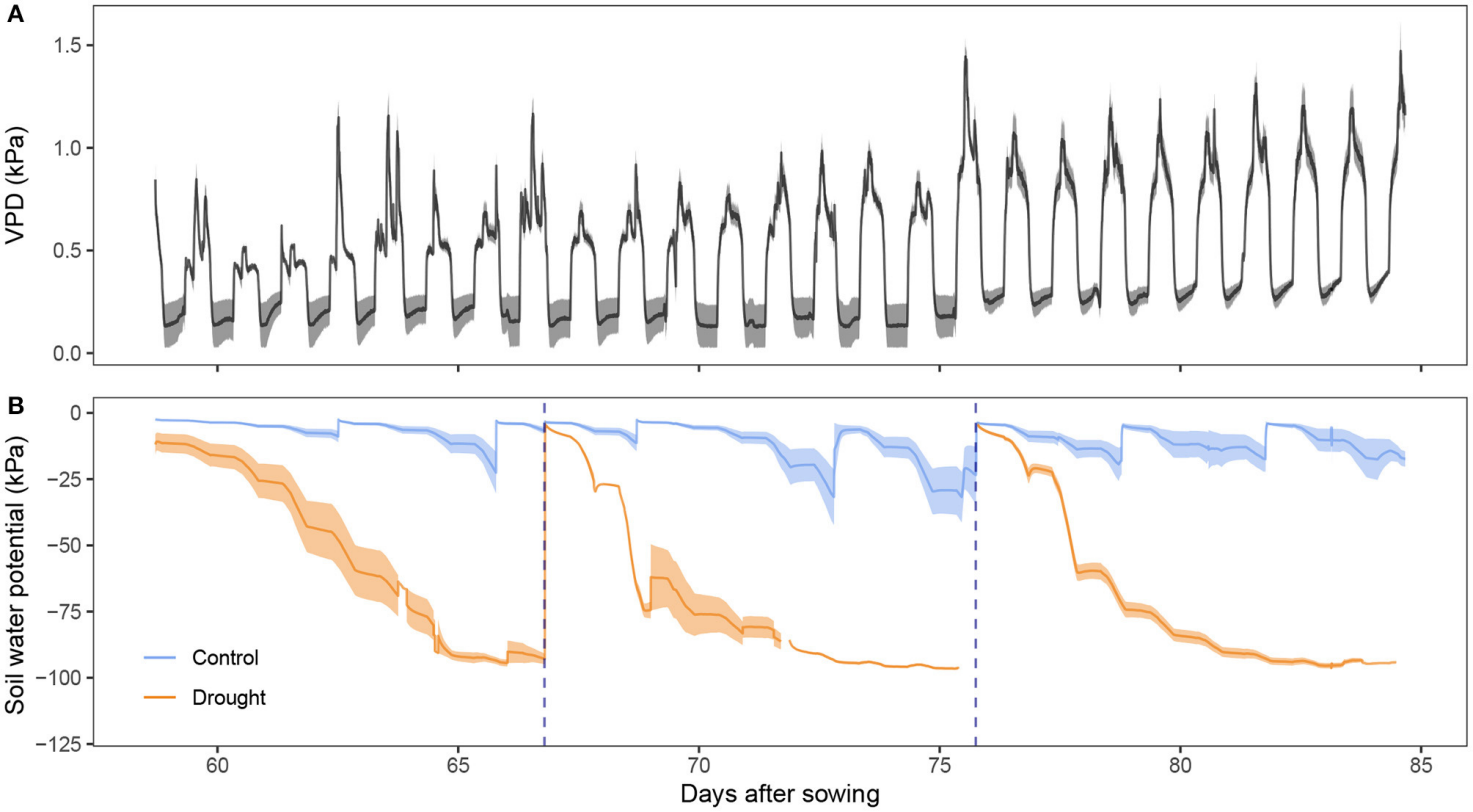
Growing environment of the wheat plants. **(A)** Vapor pressure deficit (VPD, kPa, *n* = 2) present in the growth chamber. **(B)** Soil water potential (kPa, *n* = 4) measured with tensiometers. Darker lines represent the mean while the lighter band the standard error. Rewatering events are marked with a blue dotted line.

The present study focused on assessing the physiology, and more particular the osmotic adjustment, of a drought sensitive wheat cultivar experiencing both pre- and post-anthesis drought. The plants were grown in a growth chamber which allowed control of the environment so that the conditions in the field could be simulated without much fluctuation. Figure 1A shows the vapor pressure deficit (VPD) in the growth chamber. Nighttime VPD fluctuated around 0.2 kPa and 0.3 kPa toward the end of the experiment. Daytime average VPD increased steadily from 0.5 to 1.0 kPa. The increase is mostly due to a slow decrease in relative humidity (data not shown) due to the decreasing number of transpiring plants, since plants were removed every 3 days for measurements. Around noon, VPD was highest due to an increase in temperature and radiation, simulating the influence of the sun. In Figure 1B, the stress imposed on the drought treated plants is visualized. The soil water potential reached values of –100 kPa (corresponding to 0% VWC) after withholding irrigation for a week. Values lower than –80 kPa should be interpreted with care because of the measurement range of the sensors. The true soil water potential was probably lower than –100 kPa at these points, and hence the stress on the plant more critical. The blue dotted lines indicate where the drought treatment was rewatered. The control plants also showed some fluctuation in soil water potential. However, the lowest soil water potential values still corresponded to 2–4% VWC (data not shown) and do not indicate drought stress yet.

**Figure 2:**
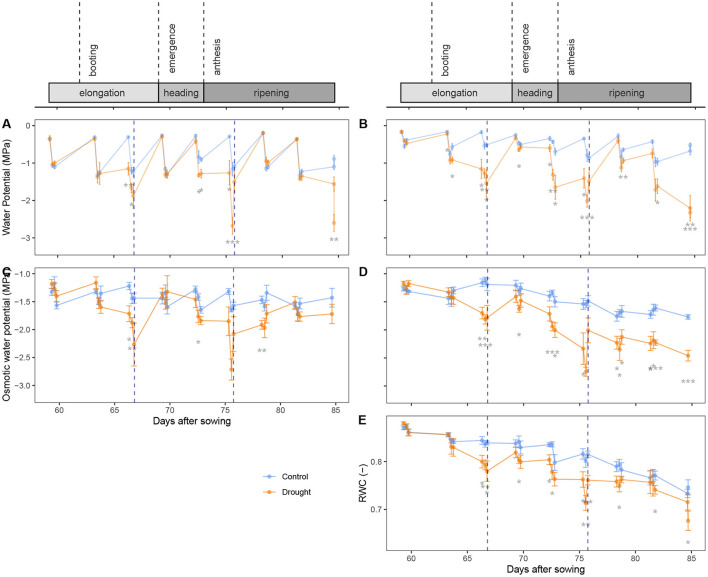
Development of different physiological variables in time, expressed as “days after sowing” (DAS): xylem water potential in the flag leaf **(A)** and bottom leaf **(B)**, osmotic potential in the flag leaf **(C)** and in the stem **(D)** and water content in the stem **(E)**. Above the graphs, different phenological periods or events are visualized on the same timeline as the data. Rewatering events are marked with a blue dotted line. The control and drought treatment are depicted in blue and orange, respectively. Every 3 days, these variables were measured at predawn, midday and afternoon. Mean (*n* = 5) and standard error are depicted and the means are connected with lines to improve the visibility of the trends. Significant differences between the control and drought treatment are indicated below the measurements. Significance levels: *p < 0.05, **p < 0.01, and ***p < 0.001.

The xylem water potential was measured in the flag (Figure 2A) and bottom leaf (Figure 2B) with a pressure chamber. There was a continuous overall decline in the water potential of the bottom leaves (Figure 2B) that is significant (β = −0.023 ± 0.0004 MPa.day^−1^, *p* = 2.63 × 10^−8^, *df* = 461, coefficient of the interaction term *DAS* × *Leaf*_*bottom*_ in Equation 3). This is probably an indication of the senescence of the bottom leaves, that gradually lost water. Predawn water potentials were close to zero as the plant was filled with water and no transpiration was occurring yet. During the day, the water potential declined in all leaves. This decline was much stronger in the flag leaves (β = 0.82 ± 0.05 MPa, *p* < 2 × 10^−16^, coefficient of the term *Time*_*predawn*_) than in the bottom leaves (β = 0.41 ± 0.12, *p* = 1.06 × 10^−8^, difference in the coefficients of *Time*_*predawn*_ and *Time*_*predawn*_ × *Leaf*_*bottom*_), as the flag leaf is most active. In the afternoon, the water potential did not change significantly any longer (*p* = 0.659, coefficient of *Time*_*afternoon*_). Drought stress had a significant impact on the water potential (*p* < 2 × 10^−16^, coefficient of *Soil*). The time points where the control and drought treatment differed significantly are marked in Figure 2. Even though the flag leaf is more active and its water potential values were generally lower, the bottom leaves showed the stress much earlier. After rewatering, the water potential was restored quickly, within 3 days, with no difference before or after anthesis.

The osmotic potential was measured in both the flag leaf (Figure 2C) and the stem (Figure 2D) with a thermocouple psychrometer. This time, not the bottom leaf, but the stem itself was sampled giving a better insight into the management of stem reserves. An overall decline can be seen in the stem (β = −0.020 ± 0.004 MPa.day^−1^, *p* = 9.54 × 10^−8^, *df* = 443, coefficient of the interaction term *DAS* × *Organ*_*stem*_ in Equation 4). This is most likely the result of stem carbohydrate mobilization (Bidinger et al., 1977; Blum, 1998), where sugars are imported into the stem as a reserve for the grain filling. The diurnal variation that was present in the xylem water potential, was less pronounced in the osmotic potential. In the morning, the osmotic potential in the flag leaves was 0.12 ± 0.05 MPa higher than at midday (*p* = 7.07 × 10^−3^, coefficient of *Time*_*predawn*_). This decline in the morning was not present in the stem (β = 0.005 ± 0.11 MPa, *p* = 0.0663, difference in the coefficients of *Time*_*predawn*_ and *Time*_*predawn*_ × *Organ*_*stem*_). The osmotic potential did not change significantly between midday and afternoon measurements (*p* = 0.544, coefficient of *Time*_*afternoon*_). Drought had a significant impact on the osmotic potential (*p* < 2 × 10^−16^, coefficient of *Soil*). The time points where the control and drought treatment differ significantly are marked in Figures 2C,D. And, as with the xylem water potential, the effects of drought on the osmotic potential were also more noticeable at the base of the plant than in its flag leaf. Figure 2C also shows that before anthesis, the osmotic potential was restored in the flag leaves within 3 days after rewatering (DAS 69). After anthesis, however, the osmotic potential was not yet fully restored 3 days after rewatering (DAS 78). In the stem (Figure 2D), the osmotic potential was only partially restored before anthesis (DAS 69), while it remained low after anthesis (DAS 78, 81 and 84).

Figure 2E shows the relative water content (RWC) in the stems of the wheat plants. There was a general decline in RWC over time that is significant (β = −0.0040 ± 0.0002 MPa.day^−1^, *p* < 2 × 10^−16^, *df* = 224, coefficient of *DAS* in Equation 5). This decline was much stronger after anthesis and is due to a strong increase in dry weight, while the absolute water content decreased (data not shown). The regression showed no significant changes in RWC in the morning (*p* = 0.278, coefficient of *Time*_*predawn*_), nor in the afternoon (*p* = 0.216, coefficient of *Time*_*afternoon*_). The effect of drought on the RWC was significant (*p* = 1.24 × 10^−15^, coefficient of *Soil*). Rewatering before anthesis slightly increased the RWC again (DAS 69), but after anthesis (DAS 78), no effect of rewatering was visible.

**Figure 3:**
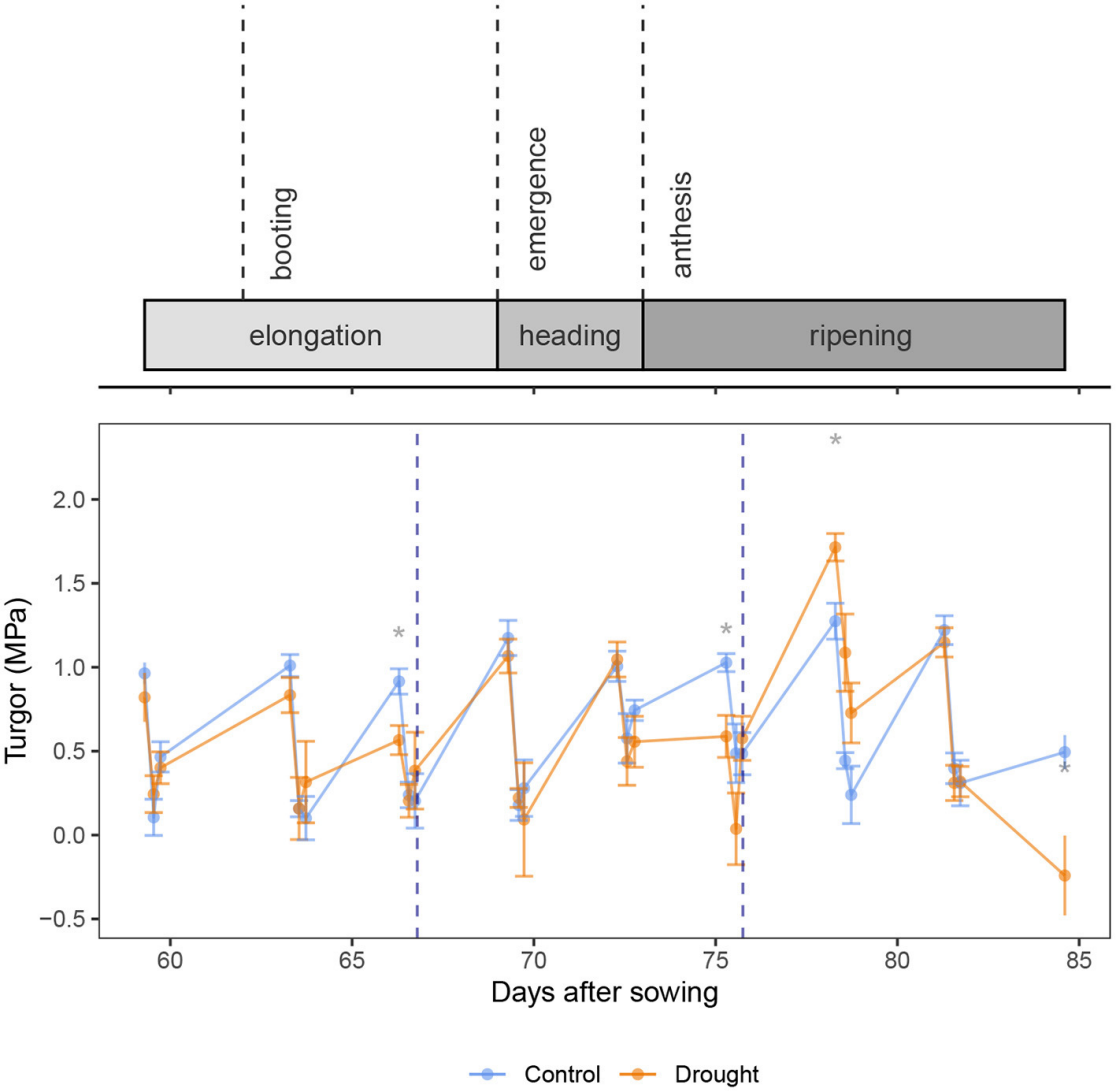
Calculated turgor (MPa) present in the flag leaves of the wheat plants. Rewatering events are marked with a blue dotted line. The control and drought treatment are depicted in blue and orange, respectively. Mean (*n* = 5) and standard error are depicted and the means are connected with lines to improve the visibility of the trends. Significant differences between the control and drought treatment are indicated above the measurements. Significance levels: *p < 0.05.

The turgor in the flag leaf (Figure 3) increased slightly over time (β = 0.016 ± 0.003 MPa.day^−1^, *p* = 2.01 × 10^−6^, *df* = 220, coefficient of *DAS* in Equation 6). After a significant decrease in the morning (β = 0.68 ± 0.06 MPa, *p* < 2 × 10^−16^, coefficient of *Time*_*predawn*_), the turgor remained unchanged in the afternoon (*p* = 0.482, coefficient of *Time*_*afternoon*_). Interestingly, drought had no significant impact on the turgor (*p* = 249, coefficient of *Soil*). When looking at the difference between the control and drought treatment at each time point (Figure 3), it can be seen that the drought treated plants had a lower turgor at predawn only when drought was severe (DAS 66 and 75). This caused wilting and led us to rewater the plants at the end of that day. Rewatering before anthesis restored the turgor to the same level as the control treatment. Rewatering after anthesis led to a significant increase in turgor in the drought treated plants (DAS 78).

**Figure 4:**
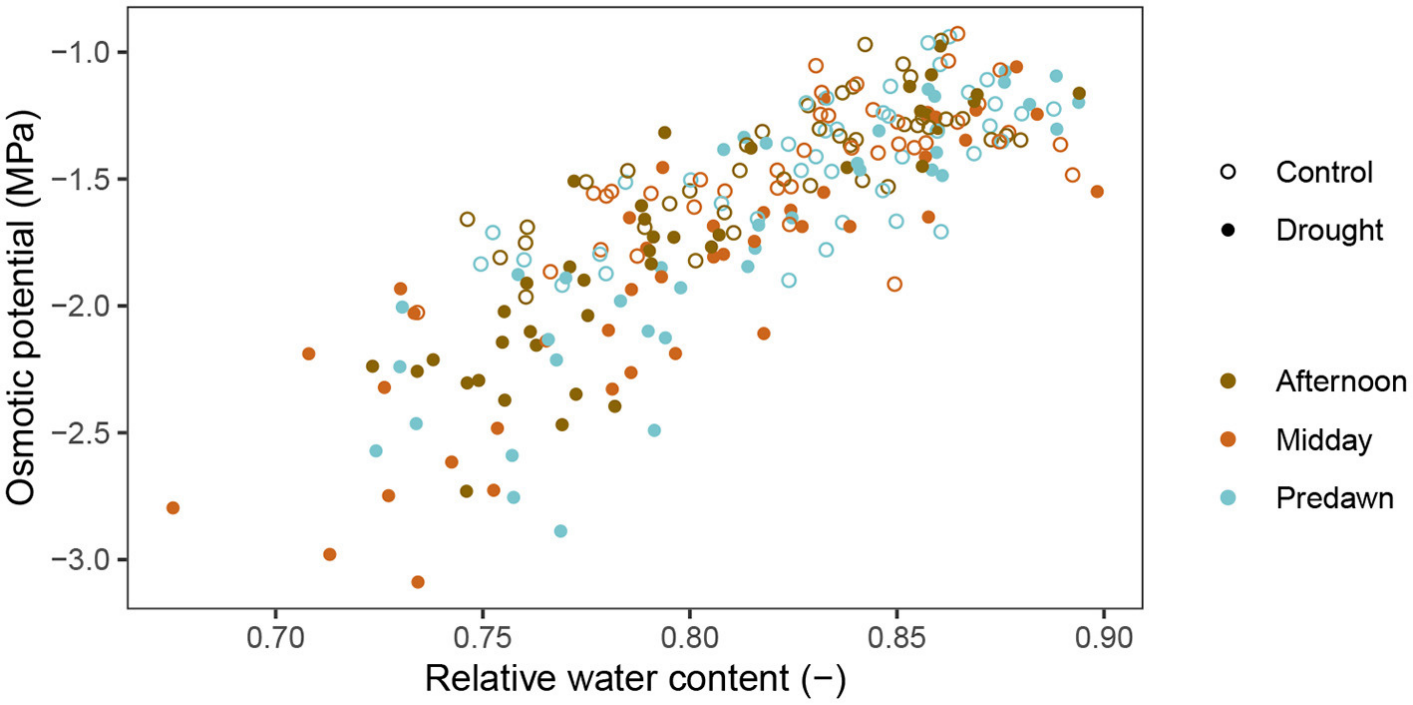
Relation between the osmotic potential and relative water content in the stems of wheat plants. The control treatment is represented by open circles while the drought treatment by full circles. Different colors represent different timeslots during the day.

**Figure 5:**
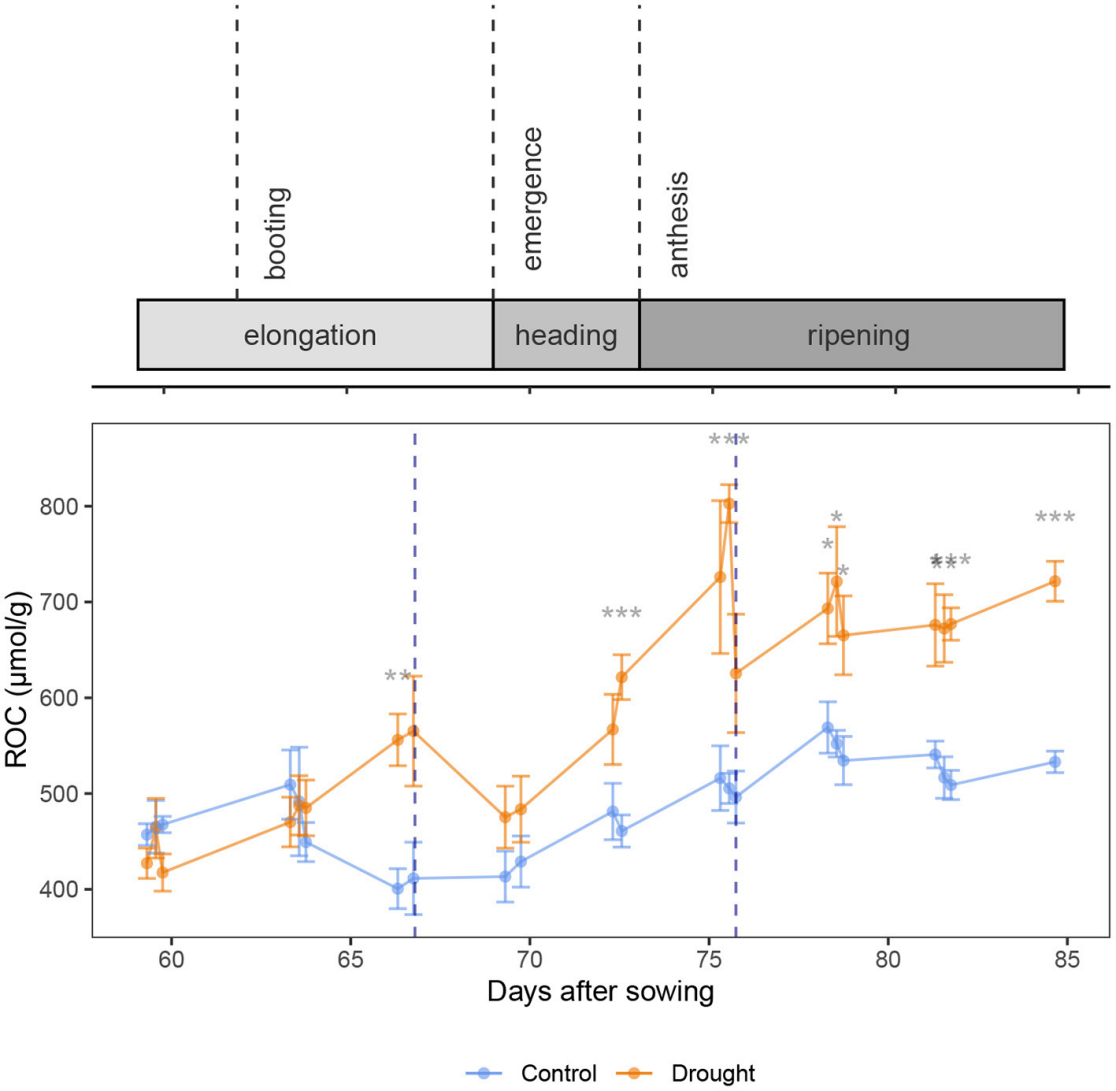
Relative Osmolyte Content (ROC) over time in wheat stems, visualizing the osmotic adjustment. Rewatering events are marked with a blue dotted line. The control and drought treatment are depicted in blue and orange, respectively. Mean (*n* = 5) and standard error are depicted and the means are connected with lines to improve the visibility of the trends. Significant differences between the control and drought treatment are indicated below the measurements. Significance levels: *p < 0.05, **p < 0.01, ***p < 0.001.

Figure 4 shows the relation between the osmotic potential and the RWC. The RWC clearly influenced the osmotic potential (β = 7.26 ± 0.35 MPa, *p* < 2 × 10^−16^, *df* = 230, coefficient of *RWC* in Equation 7). This means that most of the changes in osmotic potential can be explained by a changed RWC and not necessarily a change in osmolyte content. No clear pattern is visible when looking at the data divided in predawn, midday and afternoon measurements, which is confirmed by the regression analysis (*p* = 0.531, 0.317, 0.298, 0.216, respectively for coefficients of *Time*_*predawn*_, *Time*_*afternoon*_, *Treatment*_*drought*_ * *Time*_*predawn*_ and *Treatment*_*drought*_ * *Time*_*afternoon*_). This means that the time of day does not explain any additional differences in osmotic potential and hence, a diurnal pattern in sugar content is not present in the wheat plants. However, drought did have a significant effect (β = −0.25 ± 0.05 MPa, *p* = 5.739 × 10^−6^, coefficient of *Treatment*_*drought*_). Specifically, at the same RWC, the drought treated plants had an osmotic potential of 0.25 ± 0.05 MPa lower than the control treatment. The relative osmolyte content (ROC), as calculated in Equation 2, is visualized over time in Figure 5. This figure shows a constant increase in osmolytes in the control, which is likely due to the sugar mobilization. Under drought stress, the osmolytes are increased significantly. Rewatering before anthesis showed a quick decline to nearly the level of the control plants again at DAS 69. After anthesis, however, the osmolyte concentration remained high (DAS 78, 81 and 84).

## 4. Discussion

This work aimed to unravel whether and when osmotic adjustment takes place in wheat plants and, more specifically, how the osmotic adjustment is affected when the plants are subjected to drought. The plants were grown in a growth chamber, minimizing the day-to-day variation in the environment (Figure 1). This is convenient because that means the temporal differences in the plant variables are due to changes in the physiology of the plant and not because of environmental fluctuations. These physiological changes are driven by phenology in the control plants and by both phenology and drought stress in the drought treatment. Because of rewatering events before and after anthesis, we could differentiate between osmotic adjustment before and after the presence of the developing ear as a carbohydrate sink.

Diurnal osmotic potential patterns have been observed in a few species, e.g. in maize (Acevedo et al., 1979), barley (Koroleva et al., 2002), sorghum (Acevedo et al., 1979; Girma and Krieg, 1992), grapevine (Sancho-Knapik et al., 2016), and trees (Rada et al., 1985; Tixier et al., 2018; Gersony et al., 2020). It is thought to be a drought adaptive mechanism to maintain turgor above the threshold for stomatal closure (Rada et al., 1985). Species displaying daily osmotic adjustment increase their sugar content in the morning to build up turgor by noon (Sancho-Knapik et al., 2016). Because of the frequency of our measurements, we could study the diurnal patterns of the osmotic potential in wheat. By combining it with the xylem water potential and relative water content (RWC) of the plants, any change in osmotic potential due to changes in the water content could be identified. Thus, any differences in osmolyte content could be distinguished from changes in water content.

As the flag leaf is the most important source of photosynthesis (Evans et al., 1972; Makunga et al., 1978), it was expected that, because of a higher stomatal conductance (Del Pozo et al., 2007), the xylem water potential in the flag leaf declined significantly in the morning, and more than in the bottom leaf (Figure 2A, both before and after anthesis). This elevated negative pressure resulted in a significant decline in the RWC of the plant (Figure 2E). The osmotic potential in the flag leaf showed a significant drop in the morning as well, although less pronounced than the xylem water potential drop (Figure 2C). A drop in osmotic potential means that the osmolyte concentration increased. This can be due to either a decrease in water content, an increase in osmolyte content, or a combination of both. The severe drop in xylem water potential suggests that any change in osmotic potential could be explained by a decrease in its water content. This is confirmed by Figure 3 that shows a decline in turgor pressure in the flag leaf in the morning, which means that the osmolyte content was not increased to uphold the turgor. Figure 5 does not show any decrease during the morning either. Consequently, the photosynthesis products that were synthesized in the flag during the morning were being transported toward sinks. In the stem, the osmotic potential did not decline significantly in the morning.

In the afternoon, there was no change in activity in the wheat plants. Xylem and osmotic potential values, and thus also turgor, remained unchanged in the flag leaves, despite a high VPD present in the growth chamber. These results indicate that stomatal conductance was increased during the morning and remained constant for the remainder of the day. In the afternoon, no more additional water was lost which is shown by the xylem water potential. Also in the stem, no significant changes in water potential or water content were detected in the afternoon. When visualizing the osmotic potential relative to the RWC (Figure 4 and Equation 7), the absence of daily osmotic adjustment in the stem was confirmed. At a given RWC, the osmotic potential was the same at predawn, midday or in the afternoon. Diurnal osmotic adjustment would have resulted in variation in osmotic potential measurements that could not be explained by the RWC and the treatment alone. Our research thus shows that diurnal osmotic adjustment does not occur in wheat plants. Photoassimilates that are being produced are most likely immediately transported to sinks under well-watered conditions or even mild drought stress. During stem mobilization, the stem is the main sink (Ehdaie et al., 2006; Saint Pierre et al., 2010). In the stem, the sugars are converted to sucrose, fructans and starch (Takahashi et al., 2001; Scofield et al., 2009) to avoid osmotic potentials becoming too low. After anthesis, the ear becomes the main sink (Maydup et al., 2010).

When the plants do not experience severe drought stress, low water potentials are not favored. The plants allow a more intense use of their water storage since they maintain a high osmotic potential (Figures 2C,D). However, when drought stress becomes severe, as on DAS 66 and 75, the osmotic potential is lowered by osmotic adjustment. The xylem water potential started low at predawn (Figures 2A,B), indicating that the water storage was not refilled properly during the night. On DAS 66 the osmotic potential declined more than the xylem potential in the flag leaf (Figures 2A,C) which indicates osmotic adjustment. On DAS 75, the data in the afternoon was influenced already by the rewatering event. In the stem, the osmotic potential declined significantly as well when the plants experienced severe drought (Figure 2D). Osmotic adjustment due to drought in the stem is also confirmed by Equation 7, where the drought treatment explained a significant part of the variation in osmotic potential that could not be explained by the RWC, and Figure 5, which shows a significant increase in Relative Osmolyte Content (ROC) due to drought on several days. The low osmotic potentials enforced water flow from the soil to the stem and the leaves. Our data thus show a significant osmotic adjustment during the elongation phase and at the beginning of the grain filling due to drought.

Karamanos et al. (1983) found a strong correlation between leaf water potential and proline content in different wheat organs, confirming the osmotic adjustment during drought stress as well. Moreover, the authors found that the proline content increased more readily upon a decreasing water potential before anthesis, suggesting a quicker response and osmotic adjustment compared to after anthesis. In our experiments, the inverse was measured. Before anthesis, the osmotic potential started declining at the day of turgor loss, which was the day of rewatering (DAS 66, Figure 2), while after anthesis, the osmotic potential in the drought treated plants was already significantly lower than the control at DAS 72, which was 3 days before the turgor loss. This does not match the findings of Karamanos et al. (1983). However, it is likely that the plants recovered only partially after the first rewatering. Figure 2E shows that on DAS 69, the RWC of the drought stressed plants did not yet reach the level of the control, when the drought stress started progressing again. Our data therefore cannot clearly distinguish the response to drought before or after anthesis. The recovery after rewatering, however, does show distinct differences. Siddique et al. (2000) saw a complete recovery of the water content when the drought was limited to the vegetative stage. While the RWC in the stem of the drought stressed plants did not reach the level of the control before anthesis in our experiment (Figure 2E), the xylem and osmotic potential in de flag leaf did (Figures 2A,C). Also the xylem water potential in the bottom leaf recovered completely. Figure 5 shows a fast decline in ROC in the stem after the first rewatering as well. Our data thus show that before anthesis the recovery is quick: within 3 days, possibly faster. The second rewatering took place after anthesis. The xylem water potential was again quickly restored in the plant (DAS 78 in Figures 2A,B), which indicates a restoration of the leaf functioning. These results match the findings of Tatar et al. (2016), who found that the photosynthetic rate, gas exchange and transpiration were also restored upon rewatering at anthesis. However, the recovery of the osmotic potential in the flag leaf and the stem lags behind (Figures 2C,D, DAS 78-82). Figure 5 shows a prolonged increase in ROC in the stem after the second rewatering. After anthesis, the wheat plants thus maintain a high sugar content in the stem. This allows the plants to maintain turgor. Bramley et al. (2015) also showed that wheat is able to preserve stem turgor under drought, even better than in the flag leaf. The authors attributed this to osmoregulation with the ultimate goal of directing water predominantly to the flag leaf. Our data confirms their hypothesis and reveals that this effect is much stronger after anthesis. The osmotic potential in the flag leaf was also retained low for longer after anthesis compared to before anthesis. This is to maintain turgor with the goal of maintaining a high stomatal conductance (Ahmad et al., 2018). This led to transpiration in the flag leaf and a hence a drop in leaf xylem water potential which results in a flow of water to the flag leaf instead of the reserves in the stem. This is confirmed by Figure 2E, where the water content in the stem remains low despite a decrease in osmotic potential. Figure 3 confirms that the water reaches the flag leaf, as the turgor in the drought treated plants reaches a level significantly higher than the control (DAS 78, predawn).

A big difference in physiology before and after anthesis, is the presence of a new and important sink: the developing ear. We hypothesize that this is the main difference as to why the osmotic adjustment is maintained longer after anthesis. The developing grain requires vast amounts of sugars. These sugars mostly come from the activity of the flag leaf, the ear itself, when it is still green and photosynthesizing (Araus et al., 1991; Maydup et al., 2010), and a small portion originates from stem retranslocation or remobilization at the end of the grain filling (Bidinger et al., 1977; Blum, 1989). Under drought, the proportion of remobilization from the stem storage can increase from 10% up to 40% of the final grain dry matter (Gent, 1994). The activity of the flag leaf and the ear still remain the largest contributors to the developing grain, however. We thus hypothesize that the osmotic adjustment is more important after anthesis simply because the developing grain is a more important sink than the stem was before anthesis. This hypothesis does not coincide completely with the findings of Karamanos et al. (1983), who discovered a quicker osmotic adjustment before anthesis. Their results suggest that osmotic adjustment is more important before anthesis, when the stem is the main sink. However, they studied osmotic adjustment by measuring proline content. While proline is often linked to osmotic adjustment, it is also still under debate whether it is an adaptive trait or a response to drought stress (Ashraf and Foolad, 2005). Nonetheless, it is still possible that different strategies exist for the response to drought vs. the recovery after rewatering.

Our research gives a broad understanding in the leaf functioning and osmotic adjustment due to drought during these two important growth phases. More research is necessary to confirm our hypothesis that the plant invests more energy in osmotic adjustment during grain filling than during stem mobilization (in the elongation phase). While we only used one drought sensitive cultivar, genotypic differences are important to study in future research. The increase in proline content under drought is known to differ among genotypes (Shao et al., 2006). Differences in the capacity of osmotic adjustment between genotypes have also been observed (Zivcak et al., 2009). Moreover, not only drought will have significant effects on the physiology of the plants. Climate change will also entail increased temperatures, and not only drought events. The combined effect of these abiotic stresses have been studied in wheat (Shah and Paulsen, 2003; Porter and Semenov, 2005; Prasad et al., 2006; Pradhan et al., 2012; Kadam et al., 2014; Asseng et al., 2015). Some studies warn for a higher impact of increased temperatures (e.g. Semenov and Shewry, 2011). However, these studies all measured the physiological parameters at only a few time points, often even only at maturity. As demonstrated in this study, higher frequency measurements can distinguish differences in different growth phases.

In conclusion, wheat plants do not show daily osmotic adjustment. Under drought, sugars are being concentrated to preserve leaf functioning. Before anthesis, rewatering restored the xylem and osmotic potential quickly. After anthesis, the osmotic potential was not completely recovered in the stem and flag leaf and remained low for longer. The measurements show that water flow is prioritized to the flag leaf at the expense of the stem water reserves.

## Data Availability Statement

The raw data supporting the conclusions of this article will be made available by the authors, without undue reservation.

## Author Contributions

SV and CP-D conceived the experiment and performed the measurements together. The manuscript was written by SV, with the support from CP-D, GH, and KS supervised the project. All authors discussed the results and contributed to the final manuscript.

## Funding

This work was supported by the Research Foundation-Flanders (FWO) through the Ph.D. grant to the SV, SB Ph.D. fellow at FWO (application number 1S00920N) and the Special Research Fund (BOF) of Ghent University, Belgium, through the basic infrastructure funding 01B04515 granted to KS.

## Conflict of Interest

The authors declare that the research was conducted in the absence of any commercial or financial relationships that could be construed as a potential conflict of interest.

## Publisher's Note

All claims expressed in this article are solely those of the authors and do not necessarily represent those of their affiliated organizations, or those of the publisher, the editors and the reviewers. Any product that may be evaluated in this article, or claim that may be made by its manufacturer, is not guaranteed or endorsed by the publisher.
